# Proteomic Analysis of the Effect of *Salmonella* Challenge on Broiler Chicken

**DOI:** 10.3390/molecules27217277

**Published:** 2022-10-26

**Authors:** Adedeji Adetunji, Theresa Casey, Jackeline Franco, Devendra Shah, Yewande Fasina

**Affiliations:** 1 Department of Animal Sciences, North Carolina Agricultural and Technical State University, Greensboro, NC 27411, USA; 2 Department of Animal Sciences, Purdue University, West Lafayette, IN 47907, USA; 3 Purdue Proteomics Facility, Bindley Bioscience Center, Purdue University, West Lafayette, IN 47907, USA; 4 School of Veterinary Medicine, Texas Tech University, Amarillo, TX 79106, USA

**Keywords:** *Salmonella enteritidis*, infection, bioinformatics, spleen, quantitative proteomics, mass-spectrometry, broiler chicken

## Abstract

*Salmonella enteritidis* is a foodborne pathogen that causes high morbidity in poultry. Proteomic analysis by liquid chromatography tandem mass spectrometry (LC-MS/MS) was used to study the effects of *Salmonella* infection on spleen proteome in broiler chickens. Day-old broilers were assigned to control (CON; n = 60) or *Salmonella* challenge (CON−SE; n = 60), and gavaged with Tryptic soy agar broth or SE. A subset of chicks was euthanized on D3 and D7 (n = 4/group/day) and the spleen was removed, and rapidly frozen, subsequently proteome was measured using label-free LC-MS/MS. Protein spectra were mapped to *Gallus gallus* Uniprot database. Differentially abundant proteins (DAP; FDR < 0.05) between days and treatments were identified using ANOVA. Cecal content of *Salmonella* in CON−SE was 3.37 log_10_ CFU/g and CON were negative. Across the 16 samples, 2625 proteins were identified. Proteins that decreased in abundance between days mediated cell cycle progression, while those that increased in abundance function in cytoskeleton and mRNA processing. SE infection caused an increase in proteins that mediated redox homeostasis, lysosomal activities, and energy production, while proteins decreased in abundance-mediated developmental progression. Proteomic signatures of spleen suggest SE infection was metabolically costly, and energy was diverted from normal developmental processes to potentiate disease resistance mechanisms.

## 1. Introduction

*Salmonella enteritidis* (SE) is a foodborne pathogen that colonizes the gastrointestinal tract of chickens, leading to persistent infection [1]. Recently the incidence of SE has increased in chickens, accounting for over half of the *Salmonella* serotypes identified [2]. The high morbidity rates caused by SE infection in chickens have resulted in great economic loss to poultry producers [3]. When an infected chicken is slaughtered, harvested meat may become contaminated with feces or ruptured gut contents harboring SE. Consumption of improperly cooked contaminated poultry meat and eggs is associated with foodborne illness in humans [4].

*Salmonella* persistently infects poultry because of its high mass of lipopolysaccharide (LPS), high-density growth, and motility as well as its ability to thrive in notably harsh conditions such as high acidity and oxidative stress found in the gastrointestinal tract [5]. As a Gram-negative bacterium, SE possesses an outer protective cell wall that harbors several pathogen-associated molecular patterns (PAMPs) which stimulate inflammation in a host [5]. *Salmonella* infections are initiated through oral ingestion and can result in intestinal colonization and the dissemination of lipopolysaccharide to internal organs through circulation including the spleen [6]. 

The spleen harbors T and B lymphocytes and as the main secondary lymphoid organ, it is the center for cellular and adaptive immunity, [7] thus infections can disrupt its normal functioning [8]. Also, maturation and storage of monocytes, B cells (mediator of humoral adaptive immunity), T cells (mediator of cellular adaptive immunity), and phagocytosis occur in the spleen. Broiler chicken challenge studies aimed at understanding the pathogenesis of *Salmonella* infection showed that infection induces inflammation associated with increased production of proinflammatory cytokines and chemokines in the gut lumen [9], as well as an influx of heterophils into the gut lumen. This study utilized liquid chromatography tandem mass spectrometry (LC-MS/MS) shotgun proteomics to study the SE infection-induced changes in the spleen proteome of young broiler chickens to better understand the pathogenesis of diseases. 

## 2. Results

### 2.1. Performance Parameters and Concentration of SE in Ceca of Broiler-Chicken

The chicks in CON−SE had 5.68 and 4.17 log_10_ CFU SE/g cecal content for D3 and D7, respectively, whereas chicks in the CON group were negative for SE (Table 1) [10].

### 2.2. Correlation Coefficients and Number of Differentially Abundant Proteins between Time Points and Groups

Analysis of the relationship of protein LFQ intensity among the samples showed moderate correlation coefficients between CON and CON−SE groups (r ≥ 0.59, Supplemental Figure S1A), and strong correlation coefficients within the same group (r ≥ 0.72, Supplemental Figure S1B). Comparing D3 and D7, a moderate correlation coefficient was observed between both time points (r ≥ 0.49, Figure S1A) and a strong correlation was observed within each time point (r ≥ 0.68, Figure S1B). 

**Figure 1:**
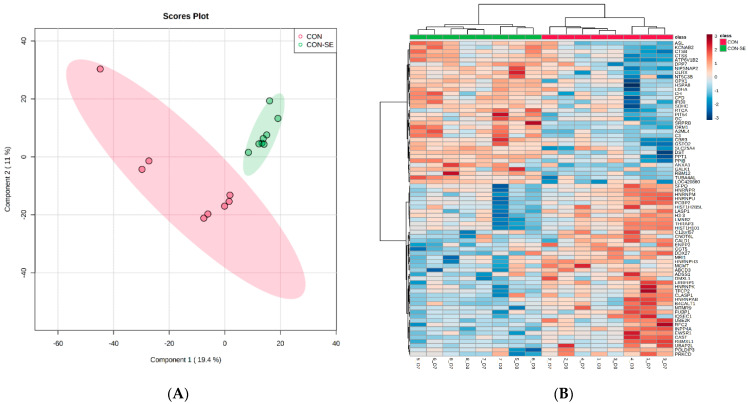
PLSDA (**A**) and heat map and dendrogram of hierarchical cluster analysis (**B**) of proteins isolated from spleen samples collected from CON and CON−SE group broiler-chickens (n = 4) on day 3 and day 7 of growth. PLSDA score plot shows distinct clusters of samples by CON and CON−SE, whereas heatmaps illustrated 75 most differentiating proteins that are greater (red) and lesser (blue) in abundance relative to CON (color-coded red) and CON−SE (color-coded green) at D3 and D7.

A search of LC-MS/MS spectra against the *Gallus gallus* Uniprot database identified 2625 proteins across the 16 samples, of which 2541 were annotated (Supplemental Table S1). Hierarchical and principal component analysis of the entire set of proteins showed that time points (D3 and D7) and treatment (CON versus CON−SE) influenced protein abundance profiles (Figure 1).

### 2.3. Differences of Protein Abundances between Time Points

There were 360 DAP (FDR < 0.05) between D3 and D7 time points across both CON and CON−SE groups (Table 2). Of these 360 proteins, 235 increased and 125 decreased in abundance between D3 and D7. Functional annotation analysis of the proteins that increased in abundance between D3 and D7 found that 13 proteins were enriched for the KEGG pathway *Splicesome*, 13 were categorized with molecular function of actin filament binding, and 31 as ATP binding (Table 3; Table S2). Among the proteins that decreased in abundance between D3 and D7 were five proteins that enriched the molecular function Heme binding, and four proteins in the KEGG pathway Cell cycle which included several subunits of members of the minichromosome maintenance protein complex (MCM) family MCM6, MCM4, MCM5, and cyclin-dependent kinase 1 (CDK1) (Table 3; Table S3).

### 2.4. Effect of Salmonella Challenge on Protein Abundance

ANOVA found 216 DAP (FDR < 0.05) between the groups (CON and CON−SE) across both time points. Overall, *Salmonella* challenge increased the abundance of 105 proteins and decreased abundance of 111 proteins (Table 2). Functional annotation analysis of the proteins that were increased in abundance in response to *Salmonella* challenge found six proteins in the KEGG pathway Biosynthesis of amino acids, six in the KEGG pathway Glutathione metabolism, four proteins categorized as response to oxidative stress, three in D-threo-aldose 1-dehydrogenase activity, eight in lysosomal activity and six proteins with molecular function of unfolded protein binding (Table 3; Table S4). Proteins less abundant in the spleen of SE-infected broiler chickens enriched actin filament binding and RNA binding (Table 3; Table S5). 

### 2.5. Differences in Proteins Abundance due to Salmonella Challenge within Day 3 and Day 7

Analysis of DAP for *Salmonella* challenge group on D3 found 106 proteins were increased in abundance and 110 proteins decreased in abundance by SE infection (Table 2). Functional annotation analysis of proteins that were greater in abundance in the spleen of SE infected broilers on day 3 included six proteins involved in the biosynthesis of amino acids, four in response to oxidative stress, six in glutathione metabolism, three in D-threo-aldose 1-dehydrogenase activity, eight in lysosome, six in apoptosis (Table 3; Table S6). Less abundant were six proteins categorized as components of the cytoskeleton, and 20 RNA binding (Table 3; Table S7). 

Analysis of DAP for *Salmonella* challenge group on D7 found 104 proteins were more abundant and 112 were less abundant in spleen of SE-infected broiler chicks (Table 2). The proteins that were less abundant in CON−SE were the same for both days 3 and 7 (Table 3; Tables S7 and S9). Although most proteins that were more abundant in spleen of CON−SE were the same for both D3 and D7 (Table 3; Tables S6 and S8), there were three unique to D3. These were KCNAB2, LOC418170, and AKR1E2, and are involved in D-threo-aldose 1-dehydrogenase activity (Table 3).

### 2.6. Effect of Salmonella Challenge on Protein Abundance

ANOVA found 216 DAP (FDR < 0.05) between the groups (CON and CON−SE) across both time points. Overall *Salmonella* challenge increased the abundance of 105 proteins and decreased abundance of 111 proteins (Table 2). Functional annotation analysis of the proteins that were increased in abundance in response to *Salmonella* challenge found six proteins in the KEGG pathway Biosynthesis of amino acids, six in the KEGG pathway Glutathione metabolism, four proteins categorized as response to oxidative stress, three in D-threo-aldose 1-dehydrogenase activity, eight in lysosomal activity and six proteins with molecular function of unfolded protein binding (Table 3; Table S4). Proteins less abundant in the spleen of SE-infected broiler chickens enriched actin filament binding and RNA binding (Table 3; Table S5). 

### 2.7. Differences in Proteins Abundance Due to Salmonella Challenge within Day 3 and Day 7

Analysis of DAP for *Salmonella* challenge group on D3 found 106 proteins were increased in abundance and 110 proteins decreased in abundance by SE infection (Table 2). Functional annotation analysis of proteins that were greater in abundance in the spleen of SE infected broilers on day 3 included six proteins involved in the biosynthesis of amino acids, four in response to oxidative stress, six in glutathione metabolism, three in D-threo-aldose 1-dehydrogenase activity, eight in lysosome, six in apoptosis (Table 3; Table S6). Less abundant were six proteins categorized as components of the cytoskeleton, and 20 RNA binding (Table 3; Table S7). 

Analysis of DAP for *Salmonella* challenge group on D7 found 104 proteins were more abundant and 112 were less abundant in the spleen of SE-infected broiler chicks (Table 2). The proteins that were less abundant in CON−SE were the same for both days 3 and 7 (Table 3; Table S7 and S9). Although most proteins that were more abundant in spleen of CON−SE were the same for both D3 and D7 (Table 3; Tables S6 and S8), there were three unique to D3. These were KCNAB2, LOC418170, and AKR1E2, and are involved in D-threo-aldose 1-dehydrogenase activity (Table 3).

## 3. Discussion

*Salmonella* infection of broiler chickens typically causes malabsorption, anemia, reduced growth rate, and inefficient feed utilization [11]. It also induces changes in immune cells and the functioning of lymphoid organs, [12] causing systemic diseases, persistent intestinal colonization, and invasion of internal organs such as the spleen [13]. To gain a better understanding of the response of animals to *Salmonella* infection in lymphoid tissue of growing broiler chickens, we evaluated the effects of SE infection on the spleen proteome during a phase of rapid growth. Although we found no effect of infection on the overall growth rate or feed efficiency of the broiler chicks over the 13 days post-infection period, [10] there was a significant effect on spleen proteome at days 3 and 7. 

Between days 3 and 7, the overall change in spleen proteome was marked by a relative decrease in the abundance of proteins that regulate DNA replication and cellular growth, [14] and an increase in the abundance of proteins that mediate post-transcriptional processing of RNA, and translocation of calcium, [15] and likely reflect that calcium regulates protein translation activities via a calcium-dependent regulatory process [16]. Proteins categorized as regulators of actin filament increased in abundance between D3 and D7 time points may potentially denote the role of actin filaments in protein translation [17]. Coincidently, transport proteins involved in cell division and the progression through the cell cycle such as multiple Minichromosome Maintenance Complex Component (MCM) proteins and CDK1 were reduced in abundance between D 3 and D7 time points. MCM plays a role in the initiation of eukaryotic genome replication [18]. Notably, CDK1 controls progression through the cell cycle and promotes the transition from G1 to S phase and G2 to M phase for mitotic onset [19]. There was also a reduction in the abundance of proteins involved in heme binding. Heme and associated enzymes are known to play vital roles in oxygen utilization for the generation of cellular energy and its reduction has been implicated in reduced cell cycle progression [20]. Overall differences in protein signatures between days 3 and 7 indicate a decrease in cell division and an increase in tissue differentiation, which was marked by increased abundance of proteins in mRNA processing and components of the cytoskeleton. 

The overall effect of *Salmonella* challenge on the proteome signature of the spleen of broiler chickens was an increase in the abundance of lysosomal proteins and proteins involved in responding to cellular stress, such as antioxidants and misfolded proteins. Following bacterial infection, the host responds to stimuli via cellular defenses that prompts the generation of free radicals [21]. This is reflected in CON−SE proteome signatures by an increase in abundance of proteins that mediate glutathione metabolism and oxidative stress. Glutathione is an antioxidant that prevents damage to important cellular components resulting from the activities of reactive oxygen species generated during cellular defense activities and oxidative stress [22]. RMM1 is associated with DNA damage repair caused by reactive oxygen species (ROS) [23]. Whereas, PRDX6, MGST1, and GPX1 catalyze the breakdown of organic hydroperoxides and hydroperoxide generated during cellular stress [24,25].

*Salmonella* challenge also increased the abundance of lysosomal proteins which are involved in the degradation of antigenic proteins and apoptotic activities [26]. Lysosomes are organelles that contain hydrolytic enzyme proteasomes, nucleases, and lipases and they degrade dead cells to ensure cellular clearance, and pathogen defense [27]. In response to the ever-changing cellular environment, lysosomal response ensures the maintenance of cellular homeostasis. Lysosomal-mediated programmed cell death can be triggered by cellular stressors such as infection and oxidative stress. In the presence of bacteria stimuli or cellular stressors, CHUK initiates activation of the NF-kappa-B signaling pathway [28]. Immune response from host cells leads to the production of inflammatory cytokines such as tumor necrosis factor-alpha (TNF-α), interleukin, and type 1 interferons (IFNs) [29]. Multiple proteins that play a role in protein folding quality control, as well as the activation of proteolysis of misfolded proteins and selection of proteins for degradation was more abundant in the spleen of *Salmonella*-infected broilers. 

Proteome signatures of the spleen of *Salmonella*-infected broilers also provided evidence for greater catabolism to support energetic needs. There was an increased abundance of proteins that play distinct roles in amino acid metabolism such as GOT1, ALDOC, ASS1, ASL, TKTL1, and GOT2. When an infection is established, energy and oxygen stores are diverted to support proliferation and activity of immune cells to fight against infections [30]. GOT1 and GOT2 catalyze the reversible transfer of an amino group between aspartate and glutamine to maintain cellular redox homeostasis [31]. ALDOC is the fourth of the Aldolase family that catalyzes reactions in the glycolysis pathway and triggers gluconeogenesis [32]. TKTL1 catalyzes reactions that link the pentose phosphate pathway to the glycolytic pathway. ASS1 and ASL play distinct roles in urea cycle ammonia removal that occur during defective glycolysis synonymous with reduced oxygen levels [33]. ALDOC, TKTL1, ASS1 and ASL proteins also perform cellular redox homeostasis maintenance functions. Thus, overall, SE infection increased the abundance of proteins involved in the innate response to foreign pathogens and clearance of antigenic proteins and infected cells as well as the triggering of gluconeogenesis to meet the high energy demands of fighting an infection. 

*Salmonella* infection of broilers decreased abundance of proteins involved in actin filament binding, RNA binding, and the cytoskeleton, indicating that SE host infection likely negatively affects the normal developmental progression of the tissue during this time. Moreover, analysis of the effect of SE infection within each day found that although proteins were highly similar, proteins involved in D-threo-aldose 1-dehydrogenase activity, including KCNAB2, LOC418170, AKR1E2 were unique to day 3. AKR-encoding genes play a role in metabolic adaptation and stress responses [34]. KCNAB2 contributes to the regulation of nerve signaling and it encodes a voltage-gated K^+^ channel β-subunit protein which are major determinants of membrane excitability [35]. To promote pathogen establishment and proliferation, intracellular bacteria undergo metabolic adaptation to the host cells in different compartments [36]. Similarly, at D3, SE induces the expression of proteins such as AKR-encoding genes that help it adapt to the host’s environment. 

**Figure 2:**
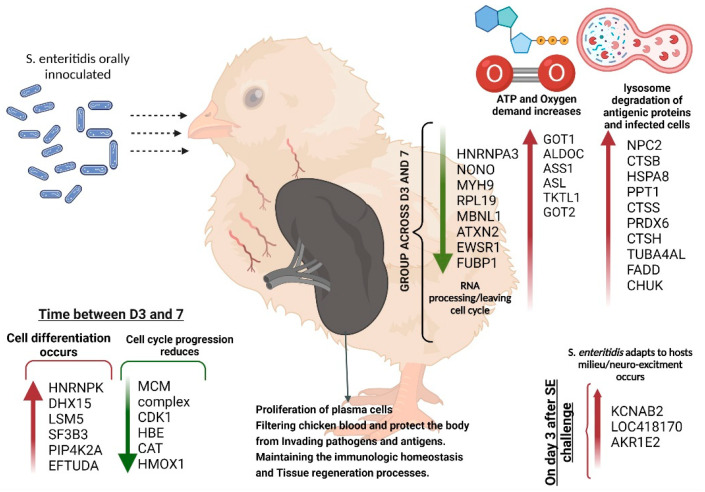
Diagram illustrating the overall signature of changes in spleen proteins between day 3 and 7 post-hatching of broilers and the impact of *Salmonella* challenge. Red arrows pointing up indicate an increase, whereas the green arrow pointing down denotes a decrease in protein abundance.

Overall, the spleen proteome of SE-infected broiler chicks indicated infection impacted redox homeostasis, increased autophagy of infected cells and the immune and inflammatory response, as well as misfolded protein binding. Proteome signatures of SE-infected chicks also indicated that normal developmental processes in the spleen of growing broilers were negatively impacted and reflected in the decreased abundance of proteins involved in post-transcriptional regulation of RNA and cytoskeletal component development (Figure 2). 

In conclusion, proteome analysis of the impact of SE infection on spleen tissue of young broiler chicks indicates that infection increases the production of energy and oxidants. Changes in the proteome between days 3 and 7 post-hatching indicate that spleen cell proliferation decreases whereas cellular differentiation increases. Together our findings indicate that although SE did not significantly impact growth performance of broiler chicks the first 2 weeks post-hatching, infection was metabolically costly, and diverted energy from normal developmental processes. 

## 4. Materials and Methods

### 4.1. Experiment Design, and Salmonella Strain used for the Experiment

Ross 708 male broiler chicks (n = 140) were obtained from a commercial hatchery at 1 day of age. The birds were vaccinated against Marek’s disease, infectious bursal, infectious bronchitis virus, and New Castle disease virus, and then transported to the Poultry Research Unit of the North Carolina A&T State University. To confirm that chicks were free of the nalidixic acid-resistant *SE* str. G1 (SE; marker strain) that was used in this challenge trial, 20 chicks were euthanized using CO_2_ asphyxiation, and ceca were aseptically removed and analyzed for *Salmonella* spp. The SE strain that was used in this challenge trial was a gift from Dr. Devendra Shah (Texas Tech University, Amarillo, TX, USA). Ceca homogenates were sequentially cultured in sterile tetrathionate (TT) and Rappaport–Vassiliadis broths (RV; Remel Inc., Lenexa, KS, USA), and xylose lysine tergitol 4 (XLT4; Becton, Dickinson and Company, Sparks, MD, USA) agar plates containing 50 μg/mL of nalidixic acid as previously described [10]. Thereafter, presumptive *Salmonella* spp. colonies were isolated and biochemically confirmed by transference into triple sugar iron (TSI; Remel Inc., Lenexa, KS) and lysine iron agar (LIA; Remel Inc., Lenexa, KS, USA) to determine fermentation end-product formation as previously described [10]. Samples biochemically confirmed as being *Salmonella* were subjected to serological latex agglutination test using polyvalent O antiserum reactive with serogroups A through I + Vi [36]. 

All remaining birds were randomly allocated into one of two groups: control (CON, n = 60) or SE challenged (CON−SE, n = 60). Each treatment consisted of four replicate pens (n = 15 birds/pen). All experimental chicks consumed an unmedicated corn–soybean meal basal starter diet throughout the study (Table 4). Birds were given access to feed and water ad libitum under a temperature of 92 °F from days 1 to 7, and at 87 °F from 8 to 14 days. Photoperiod consisted of continuous (23L:1D) lighting at 30 lux from placement for a total of 14 days. The CON−SE treatment was administered 1 mL of 7.46 × 10^8^ colony-forming units (CFU) SE/mL of inoculum (i.e., SE in Tryptic soy agar (TSA) broth) by oral gavage at 1 day of age. Birds in the CON treatment were gavaged with 1 mL of sterile TSA broth alone [37]. 

Corn–soybean meal was fed to the birds used in this experiment. Vitamin Premix, supplied per kilogram of diet: Vitamin A (6600 IU), Vitamin D (1980 IU), Vitamin E (33 IU), Vitamin B12 (0.02 mg), Biotin (0.13 mg), Menadione (1.98 mg), Thiamine (1.98 mg), Riboflavin (6.60 mg), d-Pantothenic Acid (11.0 mg), Vitamin B6 (3.96 mg), Niacin (55.0 mg), Folic Acid (1.1 mg). Mineral Premix, supplied per kilogram of diet: Manganese (Mn), 60 mg; Zinc (Zn), 60 mg; Iron (Fe), 40 mg; Copper (Cu), 5 mg; Iodine (I), 1.2 mg; Cobalt (Co), 0.5 mg. Experimental diets were analyzed for proximate nutrient composition by Eurofins Scientific Inc. Nutrient Analysis Center, 2200 Rittenhouse Street, Suite 150, Des Moines, IA 50321.

### 4.2. Growth Performance Evaluation and Spleen Sample Collection

On day 3 (CON, n = 4; CON−SE, n = 4) and 7 (CON, n = 4; CON−SE, n = 4) post-challenge, birds were randomly selected and euthanized using CO_2_ asphyxiation, then aseptically necropsied for the removal of spleen. Each spleen collected was placed in a cryovial, snap frozen in liquid nitrogen, and stored at −80 °C until time for proteomic analysis. 

### 4.3. Protein Extraction and Proteomic Analysis

Protein sample preparation, as well as shotgun LC-MS/MS analysis, were carried out at Purdue University’s Proteomics Facility in the Bindley Bioscience Center (West Lafayette, IN). Spleen samples were homogenized in 100 mM Hepes-KOH buffer at 6500 rpm for 90 s in a bead beater (Bertin Technologies SAS, Paris, France. Lysate was transferred to a new tube and protein concentration was measured using a bicinchoninic assay (BCA, Thermo Fisher Scientific, Waltham, MA, USA). Then, 50 µg of proteins from each sample was transferred to a 1.5 mL tube and dissolved in 8 M urea containing 10 mM dithiothreitol (DTT) and incubated at 37 °C for 1 h to reduce disulfide bonds. Cysteines were alkylated by incubating the sample for 1 h in the dark at room temperature (RT) with an alkylating reagent containing 97.5% acetonitrile, 0.5% triethyl phosphine, and 2% of iodoethanol. Samples were dried on a vacuum centrifuge, resuspended in 40 µL of 25 mM ammonium bicarbonate containing 2 µg of Pierce trypsin protease MS grade (Life Technologies, Carlsbad, CA, USA), and digested overnight at 37 °C. Peptide digests were desalted using MicroSpin columns (C18 silica, The Nest Group Inc., Ipswich, MA, USA). Peptides were dried in a heated vacuum centrifuge and stored at a temperature of −80 °C. LC-MS/MS samples were resuspended in 3% acetonitrile/0.1% formic acid at a final concentration of 1 µg/µL and 1 µL was used for analysis.

### 4.4. LC-MS/MS Analysis 

The Dionex UltiMate 3000 RSLC nano System (ThermoFisher Scientific, Odense, Denmark) connected to a Q Exactive HF Hybrid Quadrupole-Orbitrap MS (Thermo Fisher Scientific, Waltham, MA, USA) was used for peptide analysis as previously described [38]. Reverse-phase peptide separation was performed at a flow rate of 200 nL/min and a temperature of 40 °C using a trap column (300 µm ID × 5 mm) packed with 5 µm 100 Å PepMap C18 medium coupled to a 25 cm long × 75 µm inner diameter analytical column packed with 1.6 µm 120 Å Aurora UHPLC C18 packed emitter column (Ionopticks, Victoria, Australia). The mobile phase solvent A was 0.1% formic acid (FA) in water and solvent B was 0.1% FA in 80% acetonitrile (ACN). The loading buffer was 98% water/2% ACN/0.1% FA. Peptides were loaded into the trap column in the loading buffer for 5 min at 5 µL/min flow rate and eluted from the analytical column with a linear 75-min linear gradient of 8–27% of buffer B, then changing to 45% of B at 100 min, 100% of B at 105-min at which point the gradient was held for 7 min before reverting to 2% of B and held until 130 min for column equilibration. Furthermore, the column was washed and equilibrated using three 30 min LC gradient periods before loading the next sample. The mass spectrometer was operated in positive ion and standard data-dependent acquisition mode with a Top20 data-dependent MS/MS scan method fragmentation of precursor ion was accomplished by higher energy collision dissociation at a normalized collision energy setting of 27%. The resolution of Orbitrap mass analyzer was set to 120,000 and 15,000 at 200 m/z for MS1 and MS2, respectively, with a maximum injection time of 100 ms for MS1 and 20 ms for MS2. The dynamic exclusion was set at 50 s and the charge state was 2–7 with 2 as a default charge and mass tolerance of 10 ppm for both high and low masses. The full scan MS1 spectra were collected in the mass range of 350–1600 m/z. The Automatic Gain Control (AGC) target of 3 × 10^6^ for MS1 and 1 × 10^5^ for MS2. 

### 4.5. Data Analysis

The raw data was imported into MaxQuant software version 1.6.3.3 for peptide matching against the Uniport database of Gallus gallus reference proteome (2626 entries, downloaded from https://www.uniprot.org, January 2020 version, accessed on 20 May 2022). The parameters for the protein identification were precursor mass tolerance of 10 ppm, enzyme specificity of trypsin/Lys-C enzyme allowing up to 2 missed cleavages, oxidation of methionine (M) as a variable modification, and iodoethanol (C) as a fixed modification. Data were filtered to retain only proteins with LFQ > 0 and MS/MS (spectral counts) ≥ 2. LC-MS/MS data was made public by depositing in MassIVE (https://massive.ucsd.edu (accessed on 20 May 2022); accession number MSV000089947). 

The LFQ intensity values were used for statistical analysis to identify differentially abundant proteins (DAPs) between CON and CON−SE as well as the different time points (D3 and 7) using analysis of variance (ANOVA) with multiple corrections for false discovery rate (FDR) to identify the overall effect of treatment (group) and time on protein abundance. Before the analysis, contaminant proteins or hits of the reverse sequence were removed. Proteins considered for DAP analysis were detected in 3 out of 4 biological replicates in both experimental groups. Protein UniProt IDs were converted to Ensembl gene IDs and official gene names using the NIH Database for Annotation, Visualization, and integration Discovery (DAVID v6.8.) https://david.ncifcrf.gov/tools.jsp, accessed on 1 March 2022. LFQ values were transformed to a logarithmic scale with a base of 2. Missing values were imputed from a normal distribution (width: 0.3, down shift: 1.8). Post-hoc Student’s *t*-test analysis was used to compare treatments (CON versus CON−SE groups) within timepoints of D3 and D7 and run in MetaboAnalyst 5.0. Proteins with an FDR < 0.05 were considered differently abundant proteins (DAPs). Proteome difference between the two groups (CON and CON−SE) was visualized using partial least squares discriminant analysis (PLSDA) scores plot. DAVID, PANTHER, [39] and GeneCards, [40] databases were used for functional annotation analysis of differentially abundant proteins. 

## Figures and Tables

**Table 1:** Effect of *Salmonella* challenge on the concentration of *Salmonella enteritidis* in ceca of broiler chicken.

Log_10_ CFU/g Cecal Contents		
Treatment	Day 3 PC	Day 7 PC
CON	ND	ND
CON−SE	5.68 ± 0.36 ^a^	4.17 ± 0.18 ^b^
*p*-value	<0.0001	<0.0001

Values are presented as Mean ± Standard Error of the Mean. Mean values with different superscript letters within a column are significantly different (*p* < 0.05). CON refers to control; ND denotes not detected. Treatment CON−SE consists of chicks orally inoculated with 7.48 × 10^8^ CFU *Salmonella enteritidis* (SE)/mL *Salmonella* at 1 d of age. PC denotes Post challenge.

**Table 2:** Number of differentially abundant proteins identified using ANOVA FDR < 0.05.

Comparison	Total	Increased	Decreased
Timepoints (D3-D7)	360	235	125
CON-CON−SE	216	105	111
CONSE@D3		106	110
CONSE@D7		104	112

**Table 3:** Representative categories enriched with differentially abundant proteins between timepoints D3 and D7, overall group, CON−SE at D3 and D7.

Term	%	*p*-Value	Genes
**TIME**			
**Proteins that increased between time points (D3 and D7)**			
Spliceosome	5.5	2.2 × 10^−5^	SRSF6, EFTUD2, SF3B1, RBM25, FUS, ALYREF, SRSF7, HNRNPK, SF3A3, DHX15, SF3B3, FUSSNW1, LSM5
Actin filament binding	5.5	7.5 × 10^−5^	CORO1C, BIN1, ACTR2, FMNL1, TLN1, CAPB, TPM1, SCIN, TPM3, MYH9, ACTR2, MYO1F, HCLS1
ATP binding	13.1	1.7 × 10^−2^	DDX17, TOP1, DDX1, CAMK2D, TOP2B, DDX18, ACTR2, ATP2A3, SMC3, EHD4, UBE2N, SYK, PRKCB, SMARCA5, SWAP70, DARS, PAK2, PKN2, RPS6KA3, NSF, LONP1, VPS4B, MYH9, DHX15, CAMK2D, ATP2A2, PIP4K2A, DNAJA4, MYO1F, LOC107051177
**Proteins that decreased between time points (D3 and D7)**			
Cell cycle	3.2	1.9 × 10^−1^	MCM6, MCM4, CDK1, MCM5
Heme binding	4.0	2.4 × 10^−2^	HBE1, HBE, CAT, CYGB, HMOX1
***Salmonella* Challenge**			
**Proteins that increased** due to *Salmonella* Challenge			
Biosynthesis of amino acids	5.7	5.9 × 10^−4^	GOT1, ALDOC, ASS1, ASL, TKTL1, GOT2
Glutathione metabolism	5.7	2.5 × 10^−4^	RRM1, GSTO2, GCLC, PRDX6, MGST1, GPX1
Response to oxidative stress	3.8	6.6 × 10^−3^	GCLC, PRDX6, SLC25A4, GPX1
D-threo-aldose 1-dehydrogenase activity	2.9	6.4 × 10^−3^	KCNAB2, LOC418170, AKR1E2
Lysosome	7.6	8.0 × 10^−5^	PPT1, CTSS, PRDX6, CTSH, IFI30, HSPA8, CTSB, NPC2
Unfolded protein binding	5.7	2.4 × 10^−4^	HSPA8, CALR, CALR3, CALR, PTGES3, HSP90AA1
**Proteins that decreased** due to *Salmonella* Challenge			
Actin filament binding	5.4	1.3 × 10^−2^	NUR2L, TPM1, MYH9, LASP1, TPM4, TPM3
RNA binding	18.0	2.7 × 10^−9^	HNRNPM, POLDIP3, RBM23, RPL19, PCBP2, HNRNPAB, HTATSF1, SRSF6, SFPQ, HNRNPK, HNRNPA3, RBMXL1, NONO, RPL11, HNRNPR, HNRNPH3, HNRNPU, MBNL1, ATXN2, EWSR1, FUBP1
**Proteins increased in the CON−SE group at D3**			
Biosynthesis of amino acids	5.7	5.9 × 10^−4^	GOT1, ALDOC, ASS1, ASL, TKTL1, GOT2
Response to oxidative stress	3.8	6.6 × 10^−3^	BCLC, PRDX6, SLC25A4, GPX1
Glutathione metabolism	5.7	2.5 × 10^−4^	RRM1, GSTO2, GCLC, PRDX6, MGST1, GPX1
D-threo-aldose 1-dehydrogenase activity	2.9	6.4 × 10^−3^	KCNAB2, LOC418170, AKR1E2
Lysosome	7.6	8.0 × 10^−5^	PPT1, CTSS, PRDX6, CTSH, IFI30, HSPA8, CTSB, NPC2
Apoptosis	5.7	1.5 × 10^−2^	TUBA4AL, CTSS, CTSH, CTSB, FADD, CHUK
**Proteins decreased in the CON−SE group at D3**			
RNA binding	17.7	3.8 × 10^−9^	HNRNPM, POLDIP3, RBM23, RPL19, PCBP2, HNRNPAB, HTATSF1, SRSF6, SFPQ, HNRNPK, HNRNPA3, RBMXL1, NONO, RPL11, HNRNPR, HNRNPH3, HNRNPU, MBNL1, ATXN2, EWSR1, FUBP1
Stress fiber	4.4	5.9 × 10^−4^	TPM1, MYH9, PDLIM7, TPM4, TPM3
**Proteins increased in the CON−SE group at D7**			
Biosynthesis of amino acids	5.9	5.0 × 10^−4^	GOT1, ALDOC, ASS1, ASL, TKTL1, GOT2
Glutathione metabolism	5.9	2.1 × 10^−4^	RRM1, GSTO2, GCLC, PRDX6, MGST1, GPX1
Lysosome	7.8	6.5 × 10^−5^	PPT1, CTSS, PRDX6, CTSH, IFI30, HSPA8, CTSB, NPC2
Apoptosis	5.9	1.3 × 10^−2^	TUBA4AL, CTSS, CTSH, CTSB, FADD, CHUK
**Proteins decreased in the CON−SE group at D7**			
RNA binding	17.7	3.8 × 10^−9^	HNRNPM, POLDIP3, RBM23, RPL19, PCBP2, HNRNPAB, HTATSF1, SRSF6, SFPQ, HNRNPK, HNRNPA3, RBMXL1, NONO, RPL11, HNRNPR, HNRNPH3, HNRNPU, MBNL1, ATXN2, EWSR1, FUBP1
Stress fiber	4.4	5.9 × 10^−4^	TPM1, MYH9, PDLIM7, TPM4, TPM3

**Table 4:** Starter diet composition for the experiment.

Composition of Starter Diets (D1 to 14)	
Ingredients	Quantity
Corn (7.5% Crude protein)	51.46
Soybean meal (47.5% Crude Protein)	40.39
Poultry fat	3.64
Limestone	1.07
Mono-Dicalcium phosphate	2.03
Salt NaCl	0.40
Sodium bicarbonate	0.02
L-Lysine HCl 98%	0.13
DL-Methionine 99.0%	0.34
L-Threonine 98.5%	0.11
NCSU Poultry Vitamin Premix^2^	0.05
NCSU Poultry Mineral Premix^3^	0.20
Choline chloride 60%	0.10
Selenium Premix	0.05
**Analyzed nutrient composition**	
Metabolizable energy (Kcal/kg)	3,117
Crude Protein, %	24.63
Crude Fat, %	4.74
Crude Fiber, %	2.3
Ash, %	6.32
**Calculated nutrient composition**	
Total Sulfur Amino Acids, %	1.03
Lysine, %	1.42
Calcium, %	0.96
Available phosphorus, %	0.48

## Data Availability

Data will be made available on reasonable request.

## Supplementary Materials

The following supporting information can be downloaded at: https://www.mdpi.com/article/10.3390/molecules27217277/s1, Figure S1: Correlation coefficient between (A) timepoints D3 and D7 (B) CON and CON−SE groups; Table S1: Label free quantification of intensity of Control (CON) and Control and *Salmonella* challenge (CON−SE) at Day 3 (D3) and Day 7 (D7) post-hatching and P-value ANOVA Analysis; Table S2: Functional annotation clusters of proteins increased for timepoints between days 3 and 7; Table S3: Functional annotation clusters of proteins decreased for timepoints between days 3 and 7; Table S4: Functional annotation clusters of proteins increased in *Salmonella* challenge group; Table S5: Functional annotation clusters of proteins decreased for *Salmonella* challenge group; Table S6: Functional annotation clusters of proteins increased for *Salmonella* challenge group at day 3; Table S7: Functional annotation clusters of proteins decreased in *Salmonella* challenge group at day 3; Table S8: Functional annotation clusters of proteins increased for *Salmonella* challenge group at day 7; Table S9. Functional annotation clusters of proteins decreased for *Salmonella* challenge group at day.

## Sample Availability

Samples of the compounds are not available from the authors.
