# Supplementary figures and images for: Proteomic Analysis of the Effect of Salmonella Challenge on Broiler Chicken
Source: Molecules. 2022 Oct 26;27(21):7277. doi: 10.3390/molecules27217277 (PMC9658033; doi:10.3390/molecules27217277)

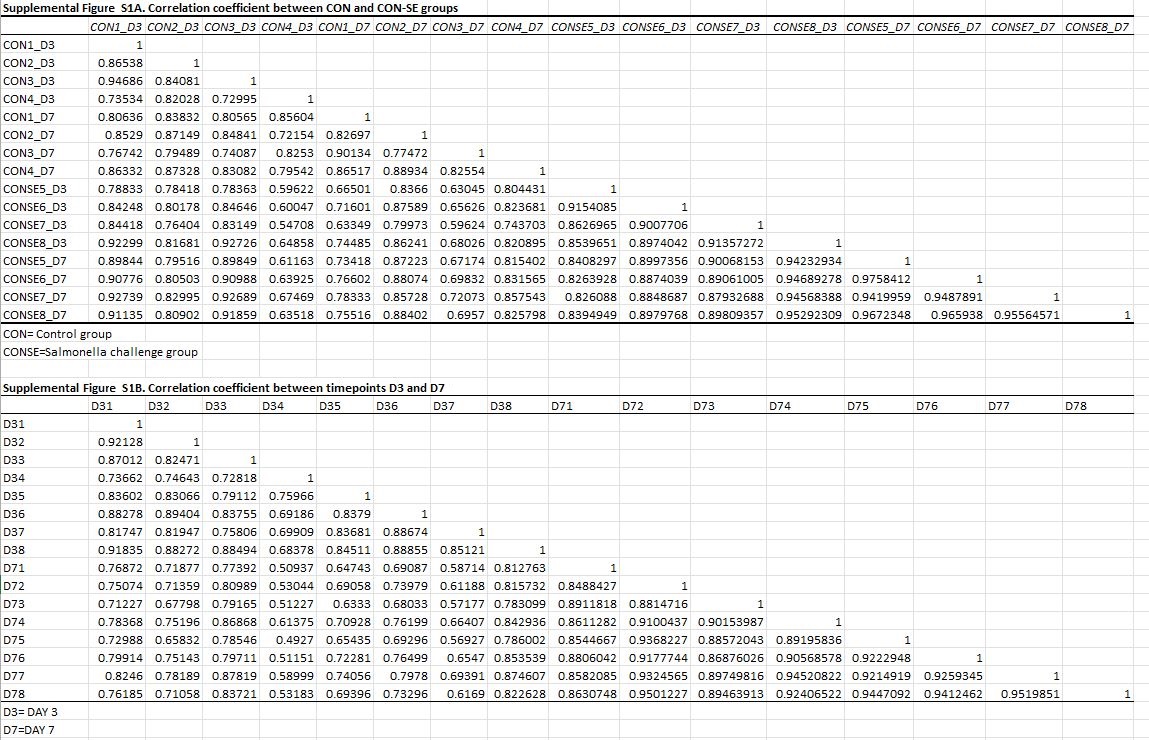

Supplement: Supplementary file 1 [file molecules-27-07277-s001.zip › Figure S1.JPG]
